# Complete thoracoscopic surgery for extensive emphysema in the right upper and middle lobes caused by right B5 bronchial atresia

**DOI:** 10.1093/jscr/rjab484

**Published:** 2021-10-26

**Authors:** Ryusuke Sumiya, Satoshi Nagasaka, Takeshi Ikeda, Yuto Suyama, Hideki Miyazaki

**Affiliations:** Department of General Thoracic Surgery, National Center for Global Health and Medicine, Tokyo, Japan; Department of General Thoracic Surgery, National Center for Global Health and Medicine, Tokyo, Japan; Department of General Thoracic Surgery, National Center for Global Health and Medicine, Tokyo, Japan; Department of General Thoracic Surgery, National Center for Global Health and Medicine, Tokyo, Japan; Pathology Division of Clinical Laboratory, National Center for Global Health and Medicine, Tokyo, Japan

## Abstract

Bronchial atresia is a rare congenital condition that may lead to infectious complications. Almost all patients with this condition are diagnosed early in life with normal lungs, making them particularly suitable candidates for thoracoscopic surgery. A 30-year-old man was referred to our hospital due to an abnormal shadow on chest radiography taken 7 years prior. Despite being diagnosed with B5 bronchial atresia, he refused to undergo surgical resection. Seven years later, he developed right chest pain. Computed tomography showed B5 bronchial occlusion, mucoid impaction and emphysematous changes. Treatment with thoracoscopic right middle lobectomy and S3 partial resection using four ports resulted in good lung expansion after discharge. This study highlights that thoracoscopic surgical resection should be considered in patients with bronchial atresia.

## INTRODUCTION

Bronchial atresia is a rare congenital occlusion of a segmental or lobar bronchus, with an estimated prevalence of 1.2 every 100 000 [1]. Surgery is the recommended treatment for bronchial atresia. If left untreated, infectious complications may arise. Almost all patients with this condition are diagnosed at a young age with normal lungs, making them particularly suitable candidates for thoracoscopic surgery [2]. Here, we present a rare case of thoracoscopic right middle lobectomy and partial resection of S3 in the right upper lobe in a patient with B5 congenital bronchial atresia.

## CASE PRESENTATION

**Figure 1:**
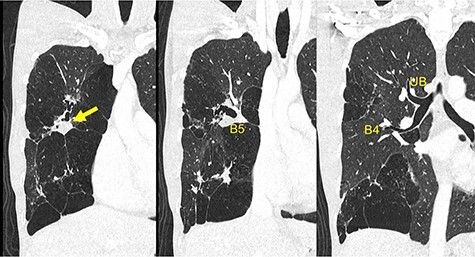
Preoperative chest CT demonstrates a mucinous impaction (Arrow) and blind-end of B5. CT, computed tomography; B, bronchus; UB, upper bronchus.

A 30-year-old man was referred to our hospital for further examination due to an abnormal shadow on chest radiography taken when he was 23 years old. Chest computed tomography (CT) revealed right B5 occlusion. Bronchoscopy showed no bifurcation in the region where the right B5 should be located. These findings are consistent with the diagnosis of bronchial atresia. Because he was not experiencing any symptoms, he refused to undergo surgical resection. Seven years later, he presented with right chest pain suggestive of an infectious complication. Antibiotic therapy resolved the infection. Following this, he was referred to our department for surgical intervention. He was a never-smoker and his medical history was unremarkable for other conditions. Laboratory examinations revealed that the patient had normal liver and renal function with no elevation of inflammatory markers (white blood cell count: 6860/μl; C-reactive protein: <0.30 mg/dl). Although carcinoembryonic antigen was within normal ranges (2.7 U/ml; normal range < 5.0 U/ml), cancer antigen 19-9 was elevated (291.8 U/ml; normal range < 37 U/ml). Chest radiography revealed an infiltration shadow with a partial hyperlucency in the right upper and lower lung fields. Chest CT showed B5 bronchial occlusion, mucoid impaction and emphysematous changes distributed throughout the right middle lobe and S3 segment of the right upper lobe (Fig. 1).

**Figure 2:**
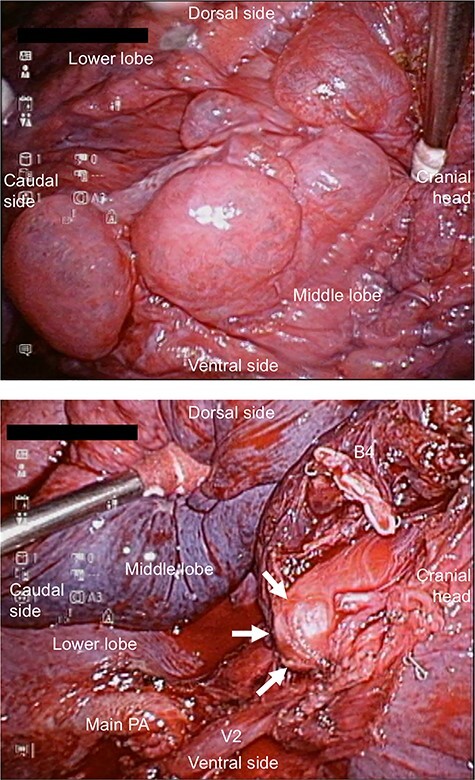
(**a**) Intraoperative view showing extensive emphysematous changes. (**b**) Intraoperative view showing the blind-end of B5. B, bronchus; PA, pulmonary artery.

**Figure 3:**
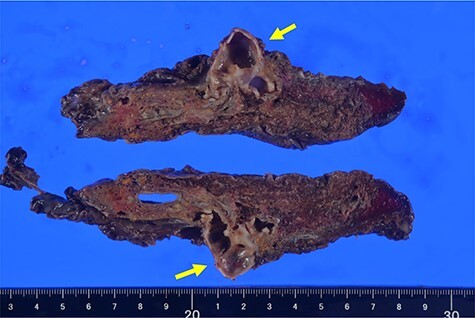
Representative image of macroscopic extracted specimen. Arrow showing blind-end of B5. B, bronchus.

The patient underwent thoracoscopic right middle lobectomy and S3 partial resection using four ports. Intraoperatively, emphysematous changes on the right middle lobe and a part of the upper lobe were observed (Fig. 2a). Moreover, although the area between the middle and lower lobes was well lobulated, the area between the upper and middle lobes was incompletely lobulated. There were no abnormalities of the pulmonary arteries or veins. The blind-end of B5 was identified (Fig. 2b). Right middle lobectomy and S3 partial resection were performed by thoracoscopic surgery. Pathologically, B5 was not connected to the bronchial tree (Fig. 3), and a dilated mucus-filled bronchus was observed. There were fibrotic and emphysematous changes in the proximal and distal side of the mucinous impaction, respectively. These findings were consistent with the pathologic diagnosis of B5 bronchial atresia. He was discharged with good lung expansion.

## DISCUSSION

Bronchial atresia, a rare congenital malformation, is different from acquired bronchial occlusion. Congenital bronchial atresia is associated with defects in vascularization during bronchial development. Radiologic and pathologic findings include blind-ending bronchus, mucoid impaction and hyperinflated lung parenchyma in the distal side of the blind-ending bronchus [2]. Congenital bronchial atresia is common in the segmental bronchi. A previous Japanese study reported that 70% of atresia occurred in the segmental bronchi [3]. The most frequent location of bronchial atresia is the left upper lobe [4].

Although congenital bronchial atresia has been described anywhere in the bronchi, congenital bronchial atresia in the right middle lobe is rare, accounting for only 3% of cases [5]. This abnormality is usually observed in the segmental bronchus. However, collateral ventilation via the Kohn’s pores causes air trapping and distension of areas downstream to the atresia, causing compression of the surrounding segment. Furthermore, air trapping and mucinous impaction can cause hyperinflation, recurrent infections and subsequent lung parenchymal expansion [6]. In our case, because radiologic and intraoperative findings revealed emphysematous changes extending from the right middle lobe to the S3 segment of the right upper lobe, right middle lobectomy and S3 partial resection were performed.

Surgical resection should be considered not only for symptomatic patients but also for incidentally discovered asymptomatic patients. The respiratory symptoms in patients with bronchial atresia are thought to be attributable to infection. Therefore, surgical resection should be considered in patients who are presenting with symptoms (e.g. dyspnea and cough) [7]. Moreover, the patients with bronchial atresia have increased risks for lung structural disruptions, drug-resistant bacterial colonization and extension of infection to the normal lung [8]. This is the reason for that surgical intervention is considered even if the patient is asymptomatic. Several surgical cases without infectious complications have also been reported (e.g. pneumothorax). Some cases with mucinous impaction were surgically resected because these were difficult to distinguish from malignant tumors [7]. In the present case, because he was symptomatic, it was considered an infectious complication due to bronchial atresia with mucinous impaction.

Although previous studies have reported that the conversion rate of thoracoscopic lobectomy for benign disease is high because of intraoperative difficulties related to inflammatory findings [9], patients with bronchial atresia are usually otherwise healthy and young with good quality lung parenchyma. Therefore, thoracoscopic surgery is a suitable less invasive approach for treatment.
